# Reinforcement of Refined and Semi-Refined Carrageenan Film with Nanocellulose

**DOI:** 10.3390/polym12051145

**Published:** 2020-05-17

**Authors:** Bakti B. Sedayu, Marlene J. Cran, Stephen W. Bigger

**Affiliations:** 1 Institute for Sustainable Industries and Liveable Cities, Victoria University, PO Box 14428, Melbourne 8001, Australia; bakti.sedayu@live.vu.edu.au (B.B.S.); stephen.bigger@vu.edu.au (S.W.B.); 2 Agency for Marine and Fisheries Research and Development, Republic of Indonesia. Jl. Pasir Putih II, Ancol Timur, Jakarta Utara 14430, Indonesia

**Keywords:** κ-carrageenan, nanocellulose fibrils, semi-refined carrageenan, biopolymer film

## Abstract

Carrageenans obtained from seaweeds can be processed into films for a range of applications including food packaging. The level of carrageenan refinement during extraction can influence the key properties, with semi-refined carrageenan (SRC) containing more impurities than the more refined carrageenan (RC). Further refinement steps, however, result in higher costs associated with the production of RC. In order to obtain a lower cost and more ecofriendly, bio-based material for food packaging applications, SRC was used in this investigation to produce a thin film reinforced with nanocellulose fibrils (NCF). Films derived from RC containing NCF were also investigated with water sensitivity and physico-mechanical and thermal properties among the properties tested. Levels of NCF were varied from 1% to 7% (*w*/*w*), and in general, the NCF reinforcement improved the overall properties of both the SRC and RC films, including the water sensitivity and moisture barrier. However, NCF inclusion in SRC film was less effective with regard to the mechanical and thermal properties compared with NCF inclusion in RC film. The enhancement in properties was attributed to the greater cohesiveness of the reinforced polymer structure and the crystalline regions formed in the structures of SRC and RC films by NCF incorporation.

## 1. Introduction

The development of polymers from seaweed-derived biomass has gained considerable interest over recent decades. When compared to biomass derived from land, seaweed-based biomass offers a more sustainable natural source that is relatively inexpensive [1]. Among the seaweed-derived polymers, carrageenan is one of the major polysaccharides that is extracted from red seaweed species and this polymer has been used for some time in a range of food applications [2]. Recently, the potential for the development of carrageenan films or composite materials has been demonstrated by several studies [3,4,5,6,7].

Commercial carrageenan is obtained from the seaweed *Eucheuma cottonii* (*Kappaphycus alvarezii*) with this type of seaweed farmed extensively in Indonesia, The Philippines, and in South-East Asia [8,9]. Processing of the seaweed involves alkali treatment to extract the carrageenan, and at this stage the product is known as semi-refined carrageenan (SRC). Further treatments such as filtration and purification processes can be applied to remove the residual substances such as cellulosic materials, thus forming a refined carrageenan (RC) [10]. Since there are fewer processing steps in the production of SRC, the global price is typically only two-thirds the price of conventional RC [11]. Refined carrageenans are widely used in the food, cosmetics, and pharmaceutical industries, whereas SRCs are mainly used in other applications, such as food packaging, that do not require a high level of refinement [12].

During the carrageenan production process, a large amount of the feedstock (*ca*. 60%–70% *w*/*w*) ends up as solid waste. More than one third of the seaweed waste biomass from the carrageenan industry (SWBC) is comprised of cellulose with a small amount of lignin (4.5% *w*/*w*) and hemicellulose (4.5% *w*/*w*) [13]. The cellulose component can be recovered from the SWBC and processed further into nanocellulose (NC) [13,14,15,16]. The NC can potentially be reutilized in the production of carrageenan or other bio-based films, and it may therefore be possible to obtain enhanced film properties in addition to gaining further environmental benefits to the carrageenan industry. Indeed, the successful recovery of cellulose from SWBC and the production of NC from seaweed have also been recently reported [13,14,15,16,17].

Carrageenan-based films show some desirable properties, particularly for the development of packaging films. However, the inherent hydrophilic nature of these films is a major issue that is responsible for the poor moisture barrier properties and low water resistance of the film [18] and this is also observed in the case of other bio-based polymer films such as those derived from starch and chitosan [19]. Since good moisture and barrier properties are required to protect and maintain many packaged foods, one technique to improve these properties involves reinforcement using nanocellulose fibrils (NCFs) [20,21,22]. Cellulose nanocrystals (CNCs) and cellulose nanowhiskers (CNWs) are also used for composite reinforcement, however, NCFs are typically longer, have a higher aspect ratio (length to diameter) and contain more amorphous regions in their molecular structure [23]. This feature can facilitate greater mechanical strength and flexibility [22] and also impart high optical clarity [24] in polymer composites. More importantly, the processing of NCFs is considerably less expensive than that of the CNCs or CNWs [25].

In synthetic polymer composites, NC inclusion in the film matrix is reported to render more ecofriendly, light-weight composites [26], whereas in bio-based polymers, NC inclusion has been reported to enhance the barrier, mechanical, and thermal properties of the resultant films [27,28]. Although previous studies have reported some positive outcomes of NC inclusion into RC films [20,29,30], no studies have been conducted using SRC to the best of our knowledge. Even though the main component in SRC, namely ĸ-carrageenan, is the same as that in conventional RC, the overall composition of these two products has shown quite distinct molecular structures, particularly in regard to the presence of insoluble components in their polymer matrices [31,32].

In view of the potential to utilize the less expensive SRC in composite film production, the present investigation aims to observe the effects of NCF reinforcement on the properties of SRC film. A comparison with RC film reinforced with NCF is presented with an emphasis on the evaluation of the physicomechanical and barrier properties.

## 2. Materials and Methods 

### 2.1. Materials

The SRC was a commercial semi-refined ĸ-carrageenan, E407a (W-Hydrocolloids Inc., Manila, The Philippines) obtained from the red seaweed species, *Eucheuma cottonii*, and full details of the SRC are given elsewhere [33]. Briefly, the SRC has the following nominal properties: <89 µm powder size; 12% (*w*/*w*) moisture content; pH 8-11, 614 kDa average molecular weight; and average composition of 50.1 mol% galactose, 43.6 mol% 3.6–anhydro-galactose, 4.3 mol% glucose, 0.9 mol% 6/4/0–O–methylgalactose, 0.9 mol% xylose, and 0.2 mol% mannose. 

The RC, type 22048 ĸ-carrageenan, was purchased from Sigma Aldrich (Sydney, Australia). This ĸ-carrageenan has a certified moisture content of 5.7% (*w*/*w*) with viscosity of 15.2 mPa s in 0.3% (w/v) water at 25 °C. Nanocellulose fibrils (NCF) were obtained from Nanocellulose Pty Ltd. (Sydney, Australia). Glycerol (99%) and KNO_3_ (≥99.0%) were purchased from Sigma-Aldrich (Sydney, Australia), and Mg(NO_3_)_2_ was purchased from Ajax FineChem (Melbourne, Australia). Milli-Q water was used in the preparation of the films and subsequent experiments.

### 2.2. Film Preparation

The SRC film preparation was performed following the method by Sedayu, Cran and Bigger [7] with some modifications. For the SRC control film, 5 g of SRC was dissolved in 250 mL of water and stirred vigorously for 15 min to dissolve the powder before heating to a temperature of 90 °C using a magnetic stirring hotplate. Glycerol was added to obtain a 40% (*w*/*w*) mixture of glycerol with respect to SRC, and the solution was stirred under heating for 30 min. The mixture was then allowed to cool for 5 min before pouring a 40 mL aliquot into a custom 200 × 150 × 3 mm acrylic casting tray. The film was allowed to dry for 36 h at room temperature (*ca*. 22 °C) before the film was peeled away from the tray for further conditioning and analysis.

To prepare the NCF incorporated SRC films, different loadings of NCF (i.e., 1%, 3%, 5%, and 7% (*w*/*w*)) relative to SRC were used. In each case, the corresponding mass of NCF was firstly dispersed in 150 mL of distilled water by vigorous stirring for 15 min followed by shearing for 10 min at 20,500 rpm using a Unidrive X1000 homogenizer (CAT, Ballrechten-Dottingen, Germany). The homogenized solution was then sonicated at 50 Hz and 80% amplitude using an ultrasonic processor UP400S (Hielscher, Teltow, Germany). Separately, for each of the formulated samples, the aforementioned SRC/glycerol solution was obtained and maintained at 90 °C before the two prepared solutions were mixed and stirred for a further 30 min before cooling and film casting.

All the RC films and RC/NCF films were prepared in accordance with the same procedures outlined above. Before all film samples were subjected to further testing, they were conditioned in a desiccator containing saturated Mg(NO_3_)_2_ for 48 h at 22 °C and 53% RH.

### 2.3. Physical Properties

A digital gauge micrometer (DML, Sheffield, UK) was used to measure the thickness of the film samples with a precision of 1 µm. A minimum of five measurements were taken and averaged, with the results later used in subsequent property calculations. Film color was measured using a CR-400 chroma-meter (Konica Minolta, Tokyo, Japan) in terms of the lightness (L*), redness-greenness (a*), and yellowness-blueness (b*). Films were placed on a standard white calibration plate (L* = 97.4, a* = 0.03, and b* = 1.77) to obtain the measurements. The opacity was measured using a UV-visible spectrophotometer (Biochrom Libra S12, Cambridge, UK) by obtaining the absorbance (λ = 550 nm) of 12.5 × 60 mm film samples placed into the instrument test cell and by dividing the absorbance by the film thickness [6,33].

### 2.4. Surface Morphology

A Hitachi Tabletop TM-3030 Plus (Hitachi, Tokyo, Japan) scanning electron microscope (SEM) was used to observe microstructural images of the surface of the films in backscattering mode using an accelerating voltage of 5 kV under a low vacuum. Prior to imaging, the samples were coated with iridium for 30 s using a Cressington 108 sputter coater (Cressington Scientific Instruments, Watford, UK). 

### 2.5. Moisture Barrier and Water Resistance Properties

The moisture content was determined gravimetrically by calculating the mass loss of a square piece of film (20 × 20 mm) after drying in an oven for 24 h at 105 °C [34]. The moisture uptake was also determined gravimetrically in accordance with the method devised by Rhim and Wang [35] using rectangular pieces of film (25 × 50 mm) that were dried for 48 h in an oven at 60 °C. Films were then placed in a desiccator containing saturated KNO_3_ (98% RH) which was incubated for 24 h at 25 °C. The moisture uptake was calculated based on the percentage mass change before and after exposure to the high humidity [33].

Water solubility was determined in accordance with the method of Rhim and Wang [35] with slight modification. Square film pieces (*ca.* 12.5 mm^2^) were firstly dried for 48 h at 105 °C before being placed in a 50 mL sample tube containing 30 mL of water. The tubes were then placed in a shaker water bath (Ratek SWB20D, Melbourne, Australia) for 30 min at 25 °C with moderate shaking. Any undissolved film was carefully removed from the tube, gently dried with tissue, then completely dried for 24 h at 105 °C. The water solubility was calculated as the percentage change in mass of the undissolved film remaining compared with the initial film mass. Water contact angle measurements were performed using a Kruss DSA30S drop shape analyzer (Kruss, Hamburg, Germany) in accordance with the method reported by Sedayu, Cran, and Bigger [33]. Water vapor permeability (WVP) was also performed in accordance with the method reported by Sedayu, Cran, and Bigger [33], based on a modified method by Sobral et al. [36].

### 2.6. Mechanical Properties

The tensile strength (TS), Young’s modulus (YM), and elongation at break (EB) of the sample films were tested using an Instron universal testing machine (Model 4301, Instron, Norwood, MA, USA) with a 5 kN load cell and in accordance with the ASTM D882 [37] method as reported by Sedayu, Cran, and Bigger [33].

### 2.7. Thermal Properties

Differential scanning calorimetry (DSC) and thermogravimetric (TG) analyses were performed using Mettler Toledo DSC1 and TGA/DSC1 instruments respectively (Mettler Toledo, Greifensee, Switzerland). The DSC measurements were performed by placing 5-10 mg of each sample into 40 µL aluminum crucibles that were heated from 50 to 320 °C at 10 °C min^−1^ under nitrogen flow (20 mL min^−1^). The TG analyses were performed by placing 8–12 mg samples in alumina crucibles that were heated from 30 to 400 °C at 10 °C min^−1^ under nitrogen flow (20 mL min^−1^). 

### 2.8. Structural Properties

The X-ray diffraction (XRD) patterns of the SRC and RC film samples were measured using a benchtop X-ray diffractometer (Miniflex 600, Rigaku, Wilmington, MA, USA). For each measurement, a rectangular piece of film was mounted on a sample glass slide and the diffraction spectrum was recorded using Cu Kα radiation (λ = 0.154 nm) at 40 kV and 20 mA over the 3–40° range. The Fourier-transform infrared (FTIR) spectra of the film samples were obtained using a Perkin-Elmer Frontier FTIR spectrophotometer coupled with a diamond crystal attenuated total reflectance accessory (Perkin Elmer, Waltham, MA, USA) over the wavenumber range 4000 to 600 cm^−1^ with 64 scans averaged for each sample.

### 2.9. Statistical Analysis

The experimental data were evaluated using an IBM SPSS Statistics 24 software package (IBM, Armonk, NY, USA) to obtain a one-way analysis of variance and to determine the significant differences among the various samples using the Duncan test at a 95% confidence level.

## 3. Results and Discussion

### 3.1. Physical and Optical Properties

**Figure 1:**
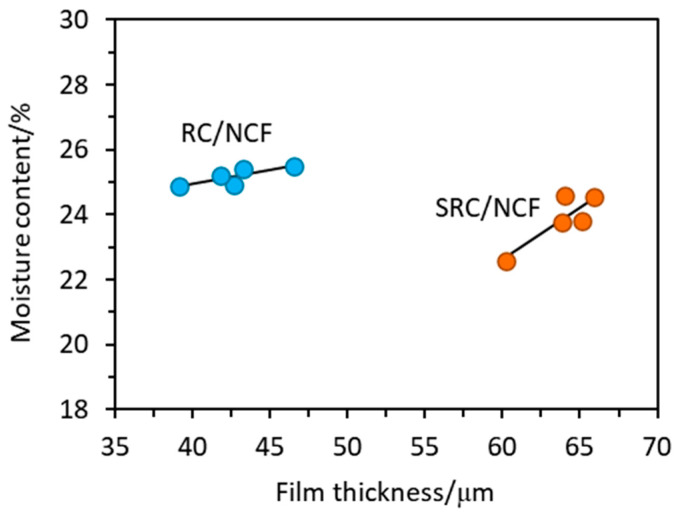
Plots of moisture content against film thickness for the SRC/NCF and RC/NCF films.

During the process of film casting, the same volumes of SRC and RC solutions were poured into the casting trays. This resulted in the formation of SRC films that were *ca*. 40% thicker than the those of the RC films (see Table 1), however, this was not unexpected since the SRC contains residual insoluble solid particles that are not present in the RC. The addition of 1% to 7% (*w*/*w*) of NCF to the SRC and RC films did not significantly affect the resulting film thicknesses with the exception perhaps of the SRC film containing 7% (*w*/*w*) of NCF, which appears to be slightly thinner than the others in its series. It is expected that the range of loadings of NCF used and/or its dimensions and compatibility in relation to the polymer matrix are such that the additive will not affect the thickness of the resultant films prepared under the conditions used in this study. As shown in Figure 1, there are slight upward trends in the plots of film thickness versus moisture content for both the RC and SRC films, and a similar observation has also been found in the case of SRC films incorporated with glycerol [7]. For the RC/NCF film samples, the inherent moisture content is approximately equal to the average of the data, *ca*. 25%, with an average film thickness of *ca*. 43 µm. Similarly, for the SRC/NCF films, the inherent moisture content is *ca*. 24% with an average film thickness of *ca*. 64 µm.

**Figure 2:**
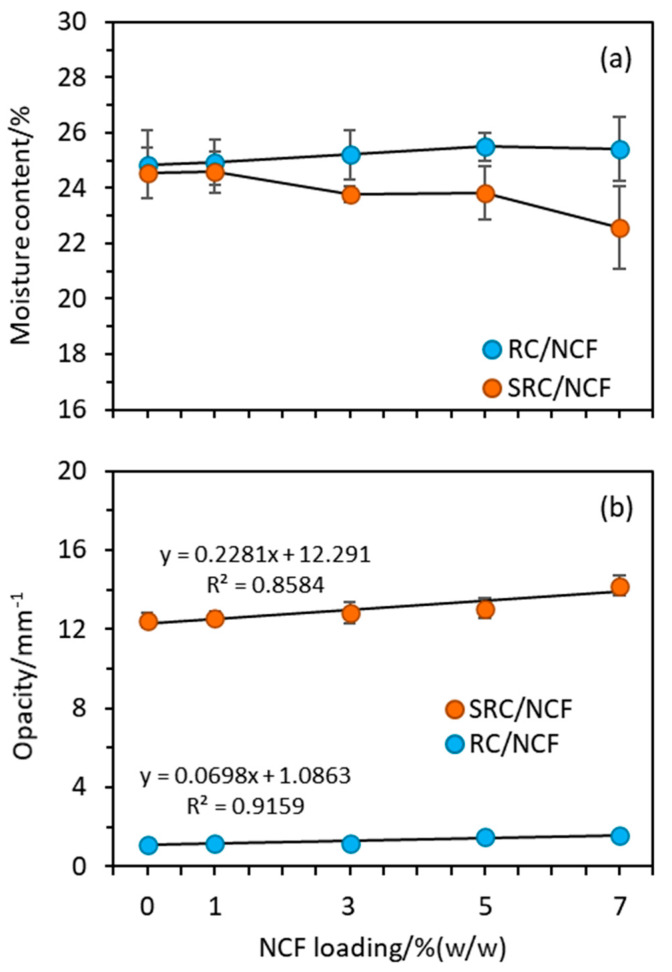
Plots of (**a**) moisture content and (**b**) opacity against NCF loading for the SRC/NCF and RC/NCF films.

Figure 2a shows plots of the moisture content against the NCF loading for the SRC/NCF and RC/NCF formulations. Interestingly, the two materials show opposite trends with the moisture content of the SRC/NCF formulation decreasing and that of the RC/NCF slightly increasing with higher NCF loadings. The downward trend in the moisture content of the SRC/NCF formulations continued over the range of NCF loadings that were tested with no threshold value being observed. The RC/NCF formulations seemed to reach a plateau at *ca*. 5% (*w*/*w*) NCF loading. The observed decrease in the moisture content of the SRC/NCF formulation may be due to the NCF displacing water molecules within the voids in the SRC structure. Conversely, the RC matrix appears to have a considerably higher inherent water content, and it is possible that the addition of NCF to it may attract further water molecules through hydrogen bonding with the NCF particles. Clearly, in the case of the SRC material it appears the latter effect is overshadowed by the propensity of the material to replace water molecules in its voids with NCF.

The film opacity plotted against NCF loading shown in Figure 2b presents the trend of apparent absorbance of the formulated films. Adding a given amount of NCF to SRC produces a slightly greater increase in the opacity of the resulting film than in the case of the RC/NCF and this may be due to the presence of residual components in the SRC/NCF formulations. The opacity in the case of both the SRC/NCF and RC/CNF formulations was further increased with the addition of higher levels of NCF due to the dispersed NCF particles obstructing the passage of light through the films. Previous investigations on other biopolymers have reported similar results in that NC distribution in the matrix has lowered film clarity [38,39,40]. In the present study, this effect may be due to the presence of the NCF particles inducing a greater extent of crystallization in the matrix of the SRC in the presence of the impurities than they do in the RC/NCF film formulation, which in turn reduces the light scattering [41,42]. 

Table 2 presents the L*, a*, and b* color values of the SRC/NCF and RC/NCF films. Similar to the opacity measurements (Figure 2b), the presence of solid residues in the SRC film formulations resulted in lower lightness values than those observed for the RC films. Unlike the RC/NCF films, which were comparatively colorless and transparent, the SRC/NCF films were more red/yellowish in appearance with higher a* and b* values that are attributable to some of the residual components in SRC such as cellulose, minerals, and insoluble aromatic compounds [43]. The incorporation of NCF into the formulation contributed to an increase in the redness of the SRC and, in general, slightly reduced the lightness of the SRC/NCF and RC/NCF films.

### 3.2. Water Sensitivity and Moisture Barrier Properties

**Figure 3:**
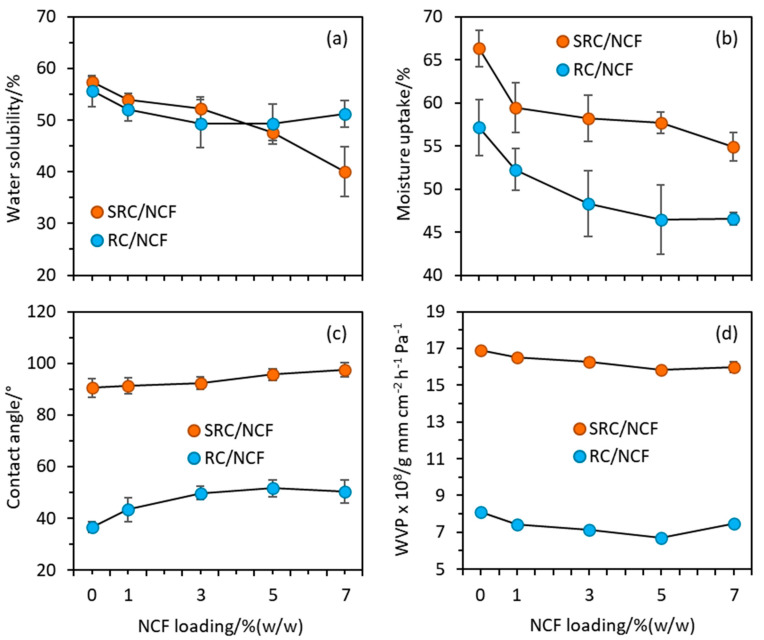
Plots of (**a**) water solubility, (**b**) moisture uptake, (**c**) contact angle, and (**d**) water vapor permeability (WVP) against NCF loading for the SRC/NCF and RC/NCF film formulations.

In food packaging applications, a certain level of water resistance is crucial to prevent the packaging material from disintegration when in contact with high moisture-containing foods such as fruits and meat [29]. Figure 3a shows the water solubility values against the NCF loading for the SRC/NCF and RC/NCF film formulations, and in general, a downward trend with increasing NCF loading is observed in the case of the SRC. However, in the case of the RC material, the downward trend appears to be halted at a threshold level of NCF (i.e., optimum loading in this case) of *ca*. 4–5% (*w*/*w*) with a possible upward trend thereafter, suggesting that agglomeration of the NCF may occur in this material at sufficiently high levels. Such agglomeration may disrupt the coherence of the polymer matrix thereby facilitating the ingress of water. The continued downward trend in water solubility at high NCF loadings observed in the case of the SRC films suggests that the NCF may be occupying voids created in the material by the impurities that are present and that a threshold loading of NCF with respect to the water solubility parameter was not reached. The reduction in water solubility by NCF incorporation may be due to the presence of relatively stable cellulose polymers that do not readily dissolve in water [44], resulting in reduced swellability and insolubility of the reinforced film in water. This may also contribute to water lower absorption [45], and similar findings have been reported for the NCF reinforcement of starch and carrageenan [21]. 

The moisture uptake data plotted in Figure 3b shows a similar downward trend with increased NCF loading for both the SRC/NCF and RC/NCF formulations, as was observed in the case of the water solubility measurements. However, the SRC/NCF formulations are inherently more hygroscopic than the RC/NCF formulations, and an equilibration in the trend of the moisture uptake values at higher NCF loadings above *ca*. 5% (*w*/*w*) is once again observed in the case of the RC/NCF formulations. There appears to be a levelling out of the moisture uptake values for the SRC/NCF commencing at *ca*. 5% (*w*/*w*) NCF loading, however, the data suggest that further improvement may be achieved at levels greater than 7% (*w*/*w*) loading.

Figure 3c shows plots of the water contact angle values against the NCF loading for the SRC/NCF and RC/NCF formulations. The SRC/NCF films exhibit consistently higher contact angle values than those of the RC/NCF formulations suggesting the surface of the SRC/NCF films are more hydrophobic. The randomly distributed impurities in the SRC that are mainly comprised of undissolved particulate cellulose and some minerals may disrupt the interaction between water and the substrate at the interface. The plot also shows a plateau in the contact angle commencing at the higher NCF loadings (*ca*. 5% (*w*/*w*)) and is therefore consistent with the data plotted for this formulation in Figure 2a.

Shown in Figure 3d are plots of the WVP values against the NCF loading for the film formulations. The WVP values of the SRC/NCF films are *ca*. two-fold greater than the values of the RC/NCF films, and this may be due to the impurities in SRC that create pores or voids which can facilitate the penetration of water molecules through the film. The data are also consistent with the higher WS, moisture uptake, and hygroscopic nature exhibited by the SRC/NCF formulations compared with the RC/NCF formulations.

Consideration of the overall water sensitivity and moisture barrier results shown in Figure 3 suggest that the incorporation of NCF into the formulations generally improved the water sensitivity of the films. Maximum effects were observed at NCF loadings up to *ca*. 7% (*w*/*w*) in the SRC polymer and at *ca*. 5% (*w*/*w*) in the case of the RC polymer. With the addition and increased loading of NCF, the moisture uptake and water solubility decreased and the contact angle increased in the case of both the SRC/NCF and RC/NCF films. These results were found to be significant (p < 0.05) in all cases at NCF levels above 1% (*w*/*w*). 

The threshold NCF loadings in the SRC/NCF and RC/NCF formulations resulted in respective decreases in the water solubility of 30% and 11%, decreases in the moisture uptake of 17% and 19%, increases in the contact angle of 8% and 40%. In addition to the improvement in the water sensitivity of SRC and RC films imparted by NCF inclusion, a decrease in the WVP was observed for both film formulations with a maximum reduction obtained at a level of 5% (*w*/*w*) NCF. This loading decreased the WVP of the SRC/NCF and RC/NCF films by *ca*. 6% and 17% respectively. The inclusion of NCF may hinder the diffusion of water molecules through the polymer matrix as a result of the tortuous blocking caused by the impermeable part of the cellulose particles in the matrix [21]. The reduction in WVP upon NCF inclusion observed in the present study is consistent with other investigations of various biocomposites utilizing NC reinforcement [19,38,46].

The enhanced properties of SRC/NCF and RC/NCF films may be further explained by the interaction between the hydroxyl groups of NCF with the hydroxyl and/or carboxyl groups in the carrageenan structure through strong hydrogen bonding, which results in an improvement in polymer cohesiveness within the matrix [47]. Similar observations have also been reported for other NC reinforced biopolymers including starch, alginate, and chitosan films [47,48,49].

An interesting finding in the present study is that the loading of NCF into neat RC was found to be optimized at 5% (*w*/*w*). Increasing the NCF above this level was ineffective at further improving the water solubility, moisture uptake, hygroscopic nature, or WVP of the RC/NCF formulation. This may be due to the possible agglomeration of the cellulose fibers in the matrix [50]. Such agglomeration has also been reported by Sánchez-García, et al. [51] upon the incorporation of fibrillated cellulose into carrageenan-glycerol polymer film at loadings above 3% (*w*/*w*). In the case of the SRC/NCF film, however, incorporation of NCF up to 7% (*w*/*w*) still showed enhancements in the water sensitivity parameters in most cases. It seems that the particulate impurities in this material interact with NCF resulting in a delayed self-agglomeration of the NCF particles in the SRC film matrix.

### 3.3. Mechanical Properties

**Figure 4:**
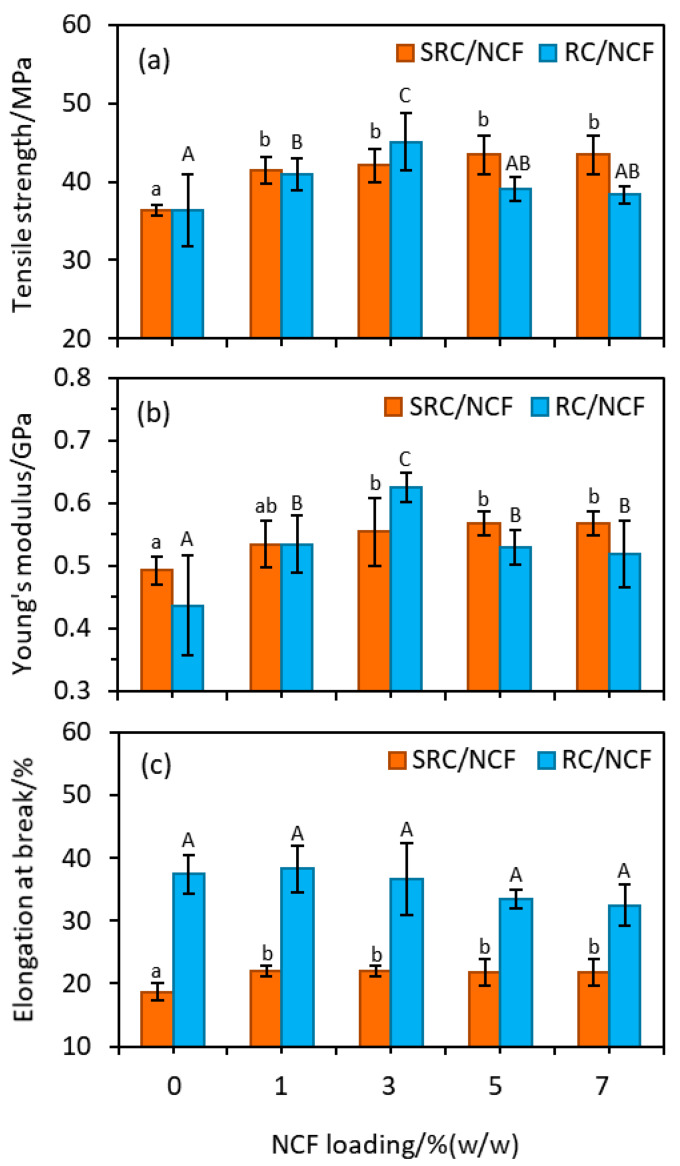
Plots of (**a**) tensile strength, (**b**) Young’s modulus, and (**c**) % elongation at break for the SRC/NCF and RC/NCF films. Values are plotted as the mean with one standard deviation error bar. Any two labelled with the same letter are not significantly different (*p* < 0.05), as determined by a Duncan test.

Food packaging films require adequate mechanical properties to prevent failure or cracking during manufacture, handling, storage, and application to food products [52]. As shown in Figure 4, the TS and YM values observed for the neat SRC films are, in general, similar to those observed for the neat RC films. The significant difference in the EB values of these materials, however, suggest that the SRC material is of considerably inferior elasticity compared to the RC material. The lower EB value may be due to the presence of the particulate impurities in the SRC that interrupt the intermolecular structure of the ĸ-carrageenan base polymer thereby weakening its cohesiveness and resulting in a less compact material compared with the RC polymer. Conversely, the particulates’ presence in the matrix of the SRC may also lead to greater stiffness and less flexibility in the produced film [40].

The incorporation of NCF into the SRC at a level of 1% (*w*/*w*) resulted in a *ca*. 20% increase in the TS of the material, which was found to be significant at the *p* < 0.05 level. Increasing the NCF loading beyond this level had no further significant effect on the TS in this case. For the RC material, the TS increased with increased loading of NCF up to a level of 3% (*w*/*w*) NCF, after which no further improvement was observed. At a level of 3% (*w*/*w*) NCF the TS of the RC material was increased by *ca*. 23%, and at higher loading the TS of this material was observed to decrease (see Figure 4).

Similar trends to those observed in the case of the TS results were also observed in the EM results for both the SRC/NCF and RC/NCF formulations, namely an initial significant increase in the EM upon the addition of the NCF followed by a plateau in the value being reached at a certain threshold level of additive. In the case of the SRC/NCF formulation, an optimal loading of 3% (*w*/*w*) NCF resulted in a *ca*. 15% increase in the EM with decreased values at higher loadings. For the RC/NCF formulation the threshold NCF level also appears to be 3% (*w*/*w*), which delivers a *ca*. 43% increase in the EM value. The trends in the TS and EM values with increased loading of NCF reflect those observed for the water sensitivity and moisture barrier properties above in that the given parameter reaches a threshold value upon the addition of NCF, after which it either reaches a plateau or, as in the case of the WS value and possibly the WVP value of the RC/NCF material, trends towards a less than desirable value.

The addition of 1% (*w*/*w*) NCF to the SRC material resulted in a *ca*. 17% increase in the EB value. At loadings greater than this, there appears to be no further improvement in this property. The addition of NCF to the RC material had no measurable effect on its EB, although there appears to be a slight decrease in the EB of this material at NCF levels above *ca*. 3% (*w*/*w*). A similar reduction in the EB value upon NCF incorporation has also been reported by Kumar and Singh [45] in the preparation of starch films.

The improvements in mechanical properties as a result of the addition of NCF may be attributed to the highly interfacial interactions between the high-strength cellulose and carrageenan polymer [53], in conjunction with an even dispersion of the cellulose particles within the polymer matrix, which subsequently strengthens the film structure [39]. Such improvement is also attributed to the high degree of compatibility between cellulose and carrageenan [54]. Beyond the threshold loadings of NCF into the SRC and RC materials, no further improvement in the mechanical properties are observed, suggesting these may be optimum levels and that higher NCF loadings may result in aggregation [20,39] or other detrimental effects. Other investigations have reported NC aggregation in ĸ-carrageenan films [20], as well as in several other biocomposite film preparations, including gelatin [55], polyvinyl alcohol [56], and alginate [47], with typical optimum NC loadings of *ca*. 5% (*w*/*w*). Furthermore, the higher NCF loading needed in the SRC formulations to reach this critical point may be due to the solid particulates in the matrix impeding the formation of aggregates.

### 3.4. Thermal Behaviour

**Figure 5:**
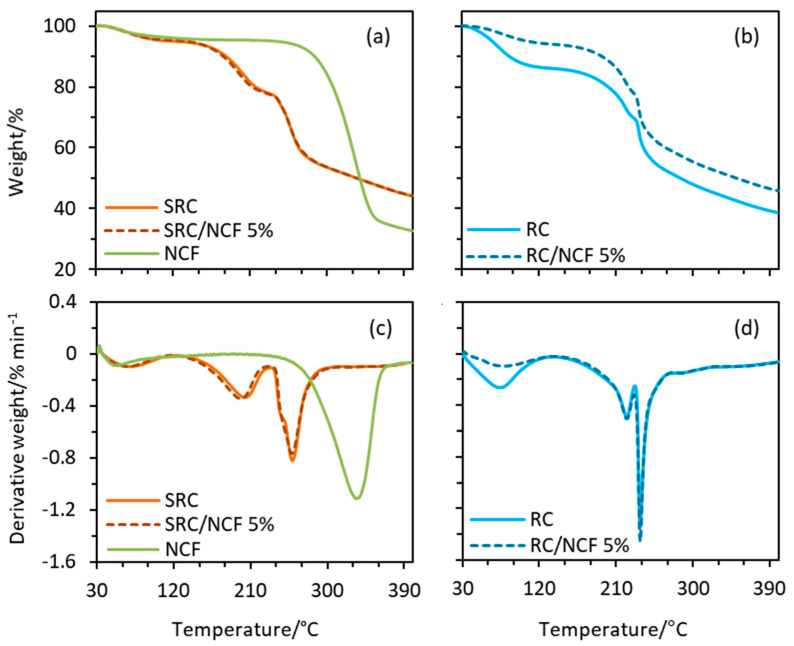
Thermogravimetric (TG) plots of normalized weight for (**a**) SRC/NCF and (**b**) RC/NCF films and derivative weight for (**c**) SRC/NCF and (**d**) RC/NCF film. Samples were heated from 30 to 400 °C at a heating rate of 10 °C min^−1^ under a nitrogen atmosphere (20 mL min^−1^ flow rate).

The thermal stabilities of the SRC/NCF and RC/NCF films were evaluated using TG analysis and the results of selected samples are shown in Figure 5 (see Figure S1 in the Supplementary Material for the full set of TG data). There are three stages of thermal decomposition of the SRC material, and the addition of NCF did not change the decomposition temperatures associated with each stage to any considerable extent with the possible exception of a slight shift to lower temperatures by *ca*. 2 °C at the peak of the second stage of decomposition (see dTG in Figure 5). The first stage of decomposition occurs between 50 and 120 °C and corresponds to the evaporation of water molecules from the film matrix. These water molecules are bound by the hydroxyl groups of the glucosyl units along the carrageenan structure and/or by the hydroxyl groups of the plasticizer (glycerol) [57]. The second stage occurs between 170 and 225 °C and corresponds to the volatilization of the plasticizer from the polymer [58]. It is followed by the third stage between 240 and 275 °C, which is attributed to the decomposition of the carrageenan polymer chains.

In the case of the RC/NCF films, the thermograms were similar across all stages of decomposition. However, the inclusion of NCF considerably delayed the mass loss during thermal decomposition, suggesting that NCF improved the thermal stability of the RC material. The mass losses of the RC/NCF film formulation during the first stage of the heating profile decreased with higher NCF loadings in the matrix of the film, and the optimum reduction of the mass loss was found at a level of 3% (*w*/*w*) NCF in RC. At the second stage of the profile (i.e., 225 °C), the 3% (*w*/*w*) NCF loading delayed the mass loss of neat RC film by 7.9%, whereas after the third stage of the profile (i.e., 400 °C), the mass loss was delayed by 7.1% (see Table S1 in the Supplementary Material). These data suggest that in the case of the RC/NCF formulations the presence of NCF retards the egress of water and glycerol from the polymer matrix and further supports the notion that the NCF thermally stabilizes the base polymer to some extent.

It can be observed from the dTG thermograms in Figure 5 that the second decomposition stage peaked at a considerably lower temperature in the case of the SRC/NCF films (*ca*. 195 °C) compared with the RC/NCF films (*ca*. 220 °C). This may suggest a stronger bonding between the glycerol plasticizer molecules and the carrageenan polymer chains in the RC matrix compared to those in the SRC matrix. The particulate impurities distributed in the SRC matrix may disrupt or impede the hydrogen bonding between the carrageenan and the plasticizer, rendering more readily volatilized plasticizer molecules. However, compared to the RC/NCF films, the SRC/NCF films demonstrated a higher peak degradation temperature at the next stage of decomposition that corresponds to the degradation of the main carrageenan polymer structure. In this case, the peak degradation temperature of the SRC/NCF films was *ca*. 257 °C whereas that of the RC/NCF films was *ca*. 236 °C. This may indicate that some impurities contained in the SRC matrix such as cellulose, minerals, or other inorganic materials have impeded the thermal degradation of the carrageenan polymer [59]. 

**Figure 6:**
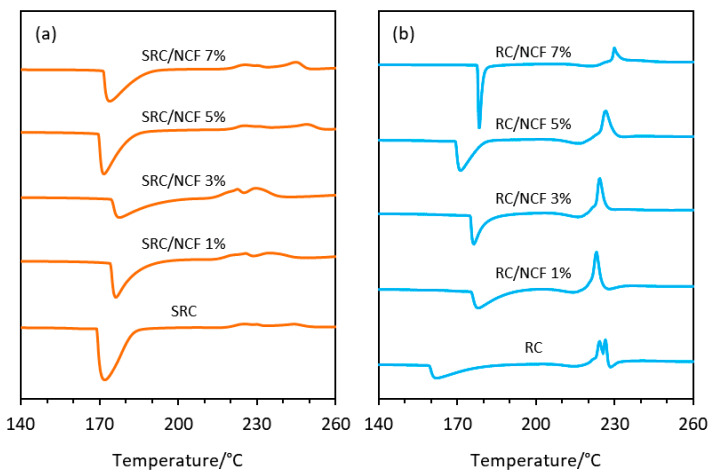
Differential scanning calorimetry (DSC) thermograms of the (**a**) SRC/NCF and (**b**) RC/NCF film formulations. Samples were heated under nitrogen (flow rate 20 mL min^−1^) from 50 to 320 °C at a heating rate of 10 °C min^−1^.

The DSC thermograms for all film formulations are shown in Figure 6, with the thermograms of the SRC and RC powder samples shown in the Figure S2 of the Supplementary Material. In general, the inclusion of NCF in the SRC polymer formulations had little effect on the thermal transitions of the material. The glass transition temperature of the neat SRC sample, which is also associated with its melting temperature, was observed to shift slightly from 172 to 178 °C at 3% (*w*/*w*) NCF loading. In the case of the RC material, a significant increase in the peak melting temperature occurred with the incorporation of 1% (*w*/*w*) NCF, where a shift from 162 to 178 °C was observed. There was no further significant increase in the peak melting temperature observed with higher NCF loadings (see Table S2 in the Supplementary Material). Furthermore, the thermal decomposition of the samples that is depicted by the exothermic peaks in the thermograms occurred over a noticeably broader range of temperatures in the SRC/NCF samples than in the RC/NCF samples. This suggests a more complex structure and subsequently a more complex degradation sequence of the SRC/NCF samples, presumably caused by the presence of impurities. In the case of the RC/NCF samples, a slight shift of the decomposition peak to a higher temperature was observed as the NCF loading in the matrix was increased. These results confirm the ability of the NCF to act as a stabilizer for this material, as suggested by the TG results shown in Figure 5.

### 3.5. Surface Imaging

**Figure 7:**
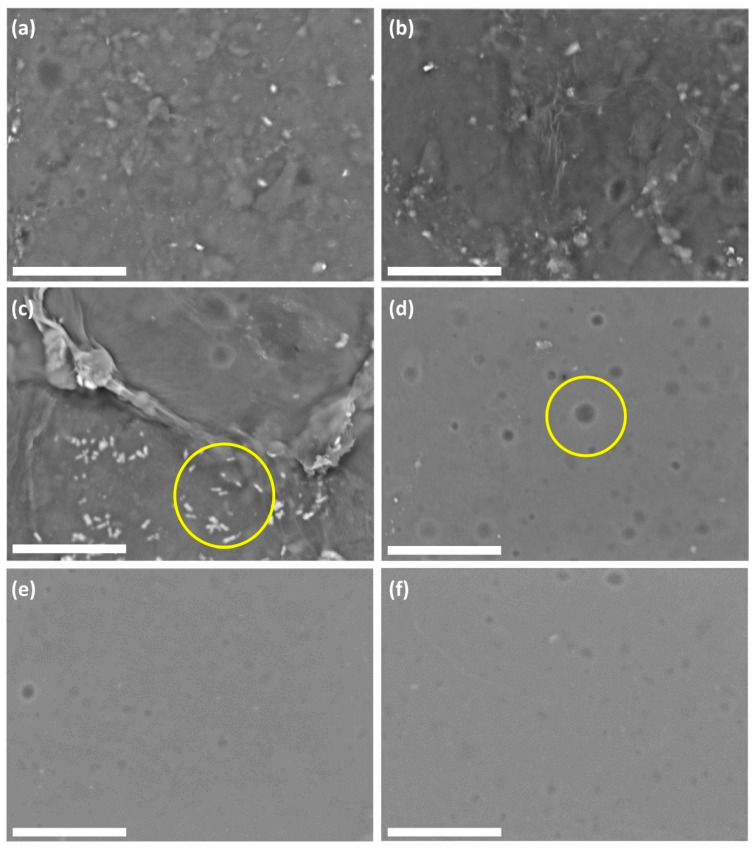
Scanning electron micrographs (2000×) of SRC reinforced with (**a**) 0%, (**b**) 3%, and (**c**) 7% (*w*/*w*) NCF, and RC films reinforced with (**d**) 0%, (**e**) 3%, and (**f**) 7% (*w*/*w*) NCF. The figure shows evidence of cellulose agglomeration (image (c)) and glycerol domains (image (d)). The images were obtained in backscattering mode using an accelerating voltage of 5 kV under a low vacuum. Scale bars represent 25 µm.

Figure 7 shows SEM images of the surfaces of the SRC/NCF and RC/NCF films containing various loadings of NCF. As expected, the SRC/NCF samples showed a rougher surface in comparison with the RC/NCF samples. The incorporation of NCF into SRC/NCF formulations up to a 3% (*w*/*w*) loading did not result in any significant changes to the film surface, but at a higher loading of 7% (*w*/*w*) NCF, a suspected cellulose agglomeration was identified in some parts of the film (see Figure 7c). The agglomeration of NCF particles at above a threshold level may explain the observed plateau or downward trend in the overall properties of the SRC film formulation, as observed previously (see Section 3.2 and Section 3.3). In the case of the RC/NCF film formulations, no noticeable changes in the film surface resulted from the inclusion of the NCF in the film matrix. The absence of impurities in the RC polymer presumably reduces the number of nucleating sites that facilitate NCF agglomeration that can contribute to the surface roughness observed in the case of the SRC material.

An interesting observation can be made in relation to the effect of NCF on the dispersion of the plasticizer (glycerol) within the RC film matrix. It appears that the addition of NCF reduces the size of the glycerol domains that are seen clearly in Figure 7d, which suggests that the presence of NCF in the matrix may increase the miscibility of the plasticizer and may prevent the aggregation of glycerol, thus facilitating a more homogenous film structure. A similar phenomenon was also reported by Sánchez-García, Hilliou, and Lagarón [51] when incorporating cellulose-nanowhiskers and glycerol in hybrid-carrageenan film. Furthermore, this behavior is also reflected in the DSC thermograms (see Figure 6) whereby the neat RC film exhibited two exothermal peaks, corresponding to two separate decomposition processes of the carrageenan polymer and glycerol, respectively. The NCF incorporated RC/NCF samples exhibit only a single exothermal decomposition peak that is indicative of a more homogeneous polymer matrix. 

### 3.6. Film Crystallinity by X-ray Diffraction

**Figure 8:**
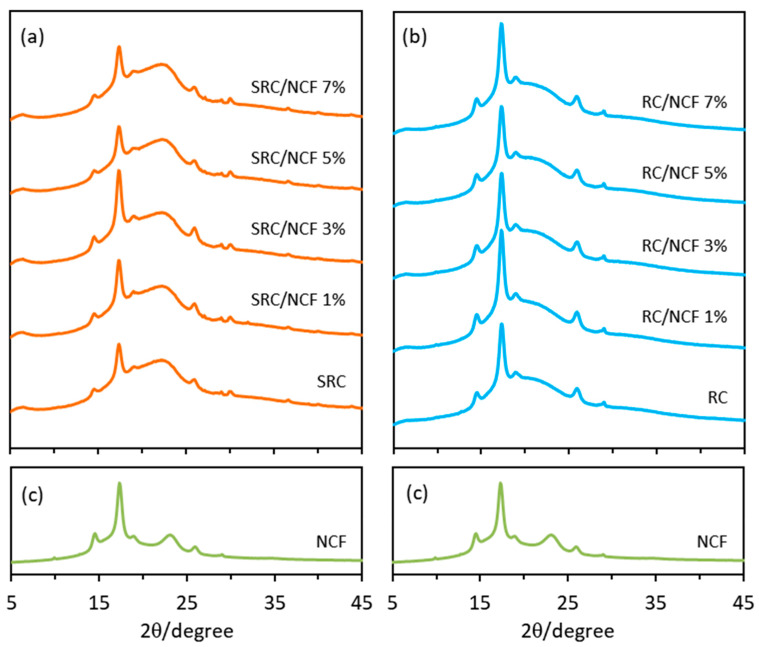
X-ray diffraction spectra of the (**a**) SRC/NCF films, (**b**) RC/NCF films, and (**c**) NCF. The diffraction spectra were recorded using Cu Kα radiation (λ = 0.154 nm) at 40 kV and 20 mA.

The XRD spectra of the SRC/NCF and RC/NCF film samples are shown in Figure 8. All spectra exhibited similar profiles at the 2θ diffraction angle and this was not unexpected since all the materials are based on a similar polysaccharide structure. The typical major peaks exhibited at 2θ = 14.5°, 17.3°, 18.9°, 26.9°, and 29.0° correspond to the predominant diffraction patterns of Type I cellulose [27] and these are also present in the NCF sample (Figure 8c). The XRD spectrum of the SRC/NCF formulations also showed a distinct broad shoulder observed in the range between 2θ = 20° to 25° indicating the presence of amorphous regions in the SRC base polymer [3], as well as some other minor peaks at around 2θ = 30° and 37° that may be due to the presence of inorganic salt residues (e.g., NaCl), other minerals, and unknown impurities found in seaweed [27,60].

The impurities in the SRC material can be present in both the amorphous and crystalline regions of the carrageenan polymer and can further influence the formation of the crystalline regions. Since there are more solid particulates in SRC compared to RC, the degree of crystallinity of the neat SRC film (56%) was found to be slightly lower than that of the neat RC film (61%). Furthermore, the degree of crystallinity is influenced by inter- and extra-molecular hydrogen bonding of the carrageenan chains in the matrix, and it can be decreased by the presence of impurities that may disrupt these hydrogen bonds. The lower crystallinity of the SRC material may therefore be caused by a reduced cohesiveness within its polymer structure. The crystalline regions of SRC/NCF and RC/NCF samples were observed to increase with an increasing level of NCF in the formulation with maximum increases in crystallinity observed for the SRC/NCF and RC/NCF formulations at 3% and 1% (*w*/*w*) NCF respectively. These levels resulted in a degree of crystallinity of 71% and 66% for the SRC/NCF and RC/NCF samples respectively (see Table S3 in the Supplementary Material). It is likely that the increased crystalline regions in the SRC/NCF and RC/NCF polymer structures contributed to the enhancement of the overall physico-mechanical properties and thermal stability in these systems [3,20], as observed elsewhere in this study. 

### 3.7. Structural Analysis by Fourier Transform Infrared Spectroscopy

**Figure 9:**
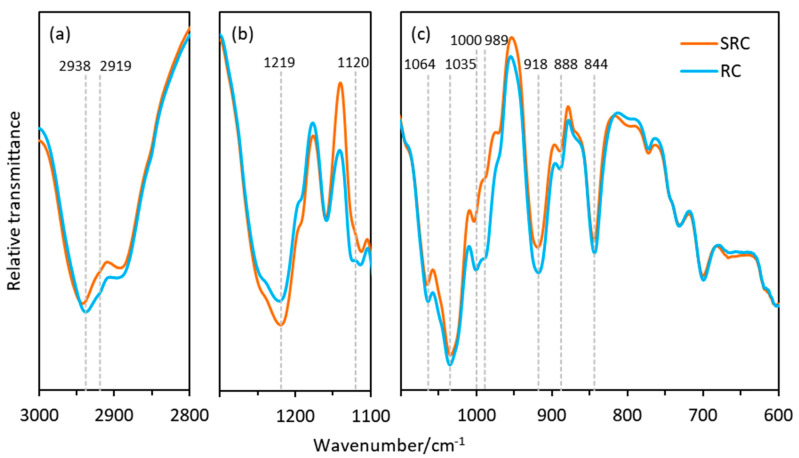
FTIR spectra of the SRC and RC films in the regions (**a**) 3000–2800 cm^−1^, (**b**) 1300–1100 cm^−1^, and (**c**) 1100–600 cm^−1^. Spectra were recorded using an average of 64 scans at 4 cm^−1^ resolution.

The FTIR spectra shown in Figure 9 depict the differences in the structures of the SRC and RC materials in selected spectral regions. In the alkane region of the RC film (Figure 9a), there is a shift in the bands at 2936 cm^−1^ and the emergence of a band at 2919 cm^−1^, which both correspond with the -CH_2_ asymmetric stretching vibrations. In the region between 1300 and 1100 cm^−1^ (Figure 9b), the RC film presents a lower intensity of the band at 1220 cm^−1^, which is assigned to the symmetric vibration of the ester sulfate O=S=O groups of carrageenan [61]. This may be a result of the additional processing steps required to produce the refined form of the carrageenan, whereby ester sulfate removal can be facilitated by alkaline treatment [62]. Moreover, the differences in the -CH_2_ peaks of the RC may facilitate the reduction of the O=S=O bending since this ester sulfate group is also associated with the amorphous structures of carrageenan polymers [61]. A new peak at 1124 cm^−1^ in the RC may also be due to a concurrent increase in C=S bonds as a result of the refining process. The region between 1100 and 600 cm^−1^ (Figure 9c) shows further differences between RC and SRC, as indicated by the peaks at 1064 and 1035 cm^−1^ (glycosidic bonds), 1000 cm^−1^ (OH-S=O bonds), 989, and 888 cm^−1^ (alkene bonds). The peaks at 918 and 844 cm^−1^ may show differences in the 3,6-AG galactose-4-sulfate and galactose-2 sulfate bonds respectively [62].

Due to the high miscibility of NCF in the SRC and RC polymer matrices, as well as the relatively low levels of NCF added in the formulations (up to 7% (*w*/*w*)), there were no discernible differences between the neat films and those containing NCF. The full set of spectra for the film samples are presented in Figures S3 and S4 of the Supplementary Material.

### 3.8. Towards Optimization of NCF Loading

Table 3 summarizes the results obtained in each of the experimental sections in terms of the threshold values that were observed for the NCF loading for the SRC/NCF and RC/NCF formulations. The table provides a means to identify a possible optimum loading of NCF in each of the SRC and RC materials that would produce the best overall performance as packaging film candidates. 

The threshold values given in Table 3 represent NCF levels where either no further improvement in the property was observed or there was a turning point in the property towards a less desirable value of its measured parameter. In the cases where no threshold was observed and/or the associated value of the measured parameter plateaued with the observed trend, these properties can be excluded from further consideration. Taking into consideration the data in Table 3 along with the trends in the individual properties with NCF loading, it is suggested that a loading of 5% (*w*/*w*) of NCF in the SRC and 3% (*w*/*w*) in the RC material will produce the optimum overall performance of these materials as candidates for packaging films.

## 4. Conclusions

An investigation of the addition of NCF as a reinforcement into SRC and RC film formulations showed general improvements in the water sensitivity and mechanical and thermal properties of these materials. The presence of natural impurities in the SRC resulted in slightly poorer optical properties of the resultant films compared with those produced with the RC material, however, the distribution of solid impurities (i.e., cellulose and minerals) within the structural matrix resulted in the SRC/NCF films having a more hydrophobic surface than the RC/NCF films. The impurities in SRC material also resulted in lower water resistance and barrier properties of SRC/NCF films compared to RC/NCF films due to the less compact matrix structure within the SRC formulation. Conversely, these impurity particulates result in the SRC/NCF films being considerably more rigid than their RC/NCF counterparts. The impurities in the SRC matrix were found to interact with NCF particles contributing a delayed self-agglomeration of the NCF particles in the SRC polymer film. The optimum levels of the NCF additive required to effectively enhance the overall properties of the SRC/NCF and RC/NCF formulations were found to be 5% (*w*/*w*) and 3% (*w*/*w*) respectively. With the exception of the reduced optical properties and the WVP values being greater than those of the RC/NCF films, the SRC/NCF films generally showed a reasonable performance compared to that found in the case of the RC/NCF film formulations, and as such, they can be further recommended as a rigid-opaque film material for food packaging applications that can be produced at significantly lower cost.

## Figures and Tables

**Table 1:** Film thickness and moisture content of the semi-refined carrageenan (SRC)/nanocellulose fibrils (NCF) and refined carrageenan (RC)/NCF films.

Film Samples	Film Thickness/µm		Moisture Content/%	
	SRC	RC	SRC	RC
NCF 0%	66 ± 4 ^a^	40 ± 7 ^A^	24.5 ± 0.9 ^a^	24.9 ± 1.2 ^A^
NCF 1%	64 ± 2 ^a^	43 ± 4 ^ABC^	24.6 ± 0.8 ^a^	24.9 ± 0.8 ^A^
NCF 3%	64 ± 3 ^a^	42 ± 6 ^AB^	23.8 ± 0.3 ^ab^	25.2 ± 0.9 ^A^
NCF 5%	65 ± 3 ^a^	46 ± 4 ^C^	23.8 ± 1.0 ^ab^	25.5 ± 0.5 ^A^
NCF 7%	60 ± 2 ^b^	43 ± 1 ^BC^	22.6 ± 1.5 ^b^	25.4 ± 1.2 ^A^

Values are given as the mean with one standard deviation. Any two means in the same column followed by the same letter are not significantly different (*p* > 0.05) as determined by the Duncan test.

**Table 2:** Color properties of the SRC and RC films.

Film Samples	L*		a*		b*	
	SRC	RC	SRC	RC	SRC	RC
NCF 0%	88.2 ± 0.2 ^a^	94.7 ± 0.2 ^A^	12.5 ± 0.4 ^a^	1.12 ± 0.04 ^A^	0.12 ± 0.03 ^a^	−0.51 ± 0.09 ^A^
NCF 1%	88.2 ± 0.2 ^b^	94.5 ± 0.2 ^B^	12.6 ± 0.3 ^a^	1.2 ± 0.1 ^AB^	0.28 ± 0.04 ^b^	−0.70 ± 0.02 ^B^
NCF 3%	88.3 ± 0.2 ^b^	94.4 ± 0.2 ^BC^	12.8 ± 0.5 ^ab^	1.2 ± 0.1 ^B^	0.23 ± 0.05 ^c^	−0.61 ± 0.25 ^AB^
NCF 5%	88.1 ± 0.4 ^b^	94.4 ± 0.1 ^B^	13.1 ± 0.5 ^b^	1.5 ± 0.1 ^C^	0.25 ± 0.04 ^c^	−0.70 ± 0.02 ^B^
NCF 7%	88.1 ± 0.4 ^b^	94.3 ± 0.1 ^C^	14.2 ± 0.5 ^c^	1.6 ± 0.1 ^D^	0.23 ± 0.03 ^c^	−0.67 ± 0.02 ^B^

Values are given as the mean with one standard deviation. Any two means in the same column followed by the same letter are not significantly different (*p* > 0.05) as determined by the Duncan test.

**Table 3:** Threshold NCF loadings for the tested properties of the SRC and RC materials.

Properties	Threshold NCF Loading/%(*w*/*w*)	
	SRC/NCF	RC/NCF
**Physical and Optical**		
Film Thickness	~7%	Note 1
Opacity	Note 2	Note 2
Crystallinity	3%	1%
Surface Texture	3% (7% max)	Note 3
**Water Sensitivity and Moisture Barrier**		
Moisture Content	Note 4	5%
Water Solubility	Note 4	4–5%
Moisture Uptake	~7%	5%
Water Contact Angle	5%	5%
Water Vapor Permeability	5%	5%
**Mechanical**		
Tensile Strength	1%	3%
Elastic Modulus	1–3%	3%
Elongation at Break	1%	3% max
**Thermal**		
Thermal Mass Loss	Note 5	3%
Melting Temperature	Note 5	1%

**Notes**
NCF loading found not to affect resultant film thickness. Opacity of SRC and RC continues to increase with increased NCF loading. No noticeable change in surface texture was observed over range of NCF loadings tested. No threshold value was observed in the case of the SRC/NCF formulation. No observable effect of NCF was found in the case of the SRC/NCF formulation.

## Supplementary Materials

The following are available online at https://www.mdpi.com/2073-4360/12/5/1145/s1, Figure S1: Complete set of TG and dTG thermographs of SRC/NCF and RC/NCF films, Table S1: Data obtained from the TG decomposition profiles of the SRC and RC films, Figure S2: DSC thermographs of SRC and RC powder, Table S2: Data obtained from the DSC thermograms of the SRC/NCF and RC/NCF films, Table S3: Degree of crystallinity of SRC/NCF and RC/NCF films, Figure S3: FTIR spectra of the SRC/NCF films, Figure S4: FTIR spectra of the RC/NCF films.
