# Supplementary material for: Reinforcement of Refined and Semi-Refined Carrageenan Film with Nanocellulose
Source: Polymers (Basel). 2020 May 17;12(5):1145. doi: 10.3390/polym12051145 (PMC7285119; doi:10.3390/polym12051145)

Reinforcement of Refined and Semi-Refined Carrageenan Film with Nanocellulose

Bakti B. Sedayu, Marlene J. Cran, Stephen W. Bigger

Supplementary Material

Figure S1. Complete set of TG (upper) and dTG (lower) thermographs of SRC/NCF (left) and RC/NCF (right) films

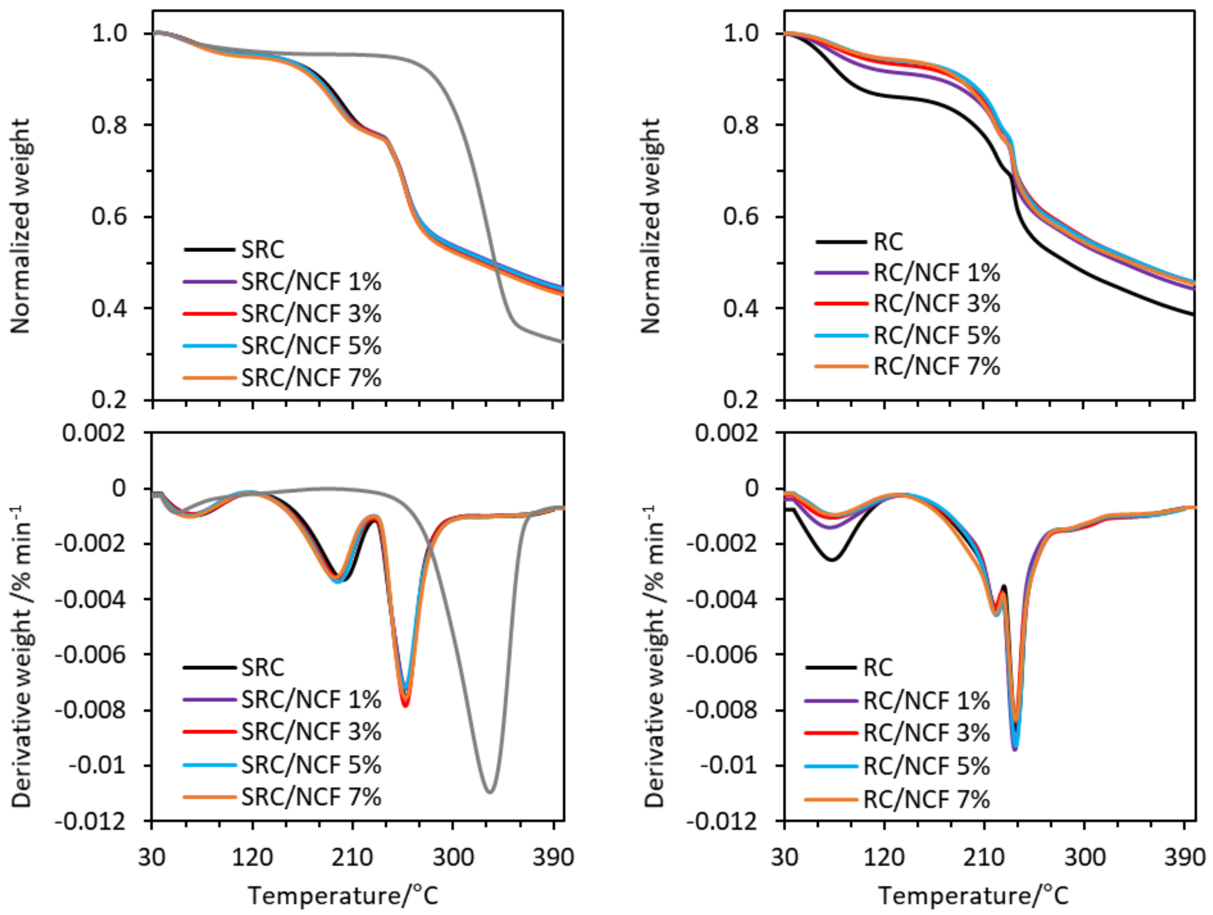

Table S1. Data obtained from the TG decomposition profiles of the SRC and RC films

Film samples	2nd decomposition peak/°C	Onset temp./°C	Endset temp./°C	Residue at 225°C/%	Max. decomposition peak/°C	Onset temp./°C	Endset temp./°C	Residue at 400°C/%
SRC	200.11	170.77	214.21	78.73	257.87	245.6	268.99	44.11
SRC/NCF1%	193.5	164.46	208.51	78.8	257.61	245.16	269.71	44.54
SRC/NCF3%	195.48	166.89	210.14	78.68	257.67	245.6	268.53	43.76
SRC/NCF5%	195.21	166.07	209.69	78.35	256.71	244.7	269.27	44.27
SRC/NCF7%	192.85	164.09	207.74	78.27	257.46	245.28	270.19	43.02
RC	219.58	197.42	224.67	71.63	235.81	243.34	239.76	38.68
RC/NCF1%	219.73	200.54	223.97	77.88	235.38	242.01	238.61	44.27
RC/NCF3%	219.8	199.71	224.07	79.49	234.95	244.19	239.06	45.74
RC/NCF5%	221.1	201.13	225.35	80.07	236.82	244.7	240.23	45.76
RC/NCF7%	220.21	193.03	225.17	78.04	236.15	244.98	240.16	45.37
NCF					335.83	303.14	353.48	32.68

Figure S2. DSC thermographs of (a) SRC and (b) RC powder

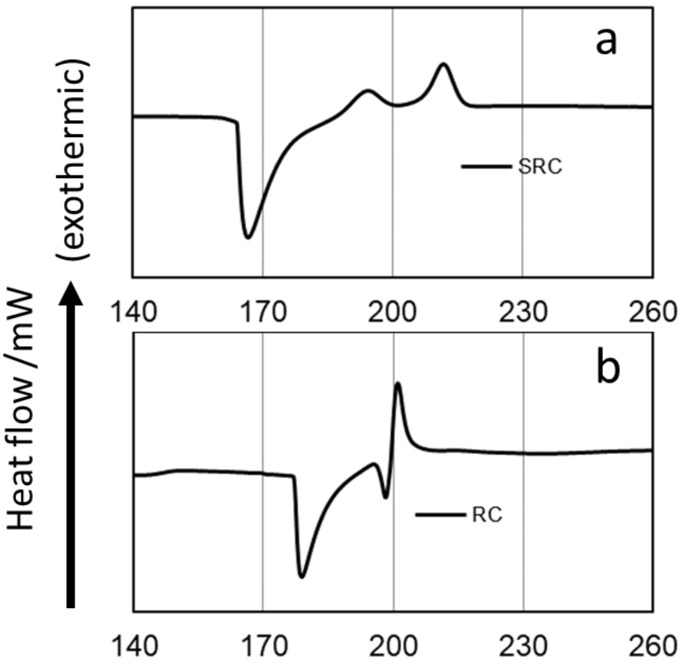

Table S2. Data obtained from the DSC thermograms of the SRC/NCF and RC/NCF films

Samples	T_g/°C	Enthalpy/mJ	Decomposition peak/°C
SRC/NCF 0%	172	-2516.09	
SRC/NCF 1%	176	-447.07	
SRC/NCF 3%	178	-450.05	
SRC/NCF 5%	172	-492.34	
SRC/NCF 7%	175	-902.07	
RC/NCF 0%	162	-336.34	224.72
RC/NCF 1%	178	-295.79	223.77
RC/NCF 3%	176	-174.52	224.76
RC/NCF 5%	171	-316.78	223.14
RC/NCF 7%	178	-398.13	230.61
NCF	153.16	219.38	>260

Table S3. Degree of crystallinity of SRC/NCF and RC/NCF films

Samples	% Crystallinity
**SRC**	56.05
**SRC/NCF 1%**	63.82
**SRC/NCF 3%**	71.20
**SRC/NCF 5%**	70.20
**SRC/NCF 7%**	55.09
**RC**	60.53
**RC/NCF 1%**	66.18
**RC/NCF 3%**	64.63
**RC/NCF 5%**	60.05
**RC/NCF 7%**	60.92
**NCF**	78.06

Figure S3. FTIR spectra of the SRC/NCF films: overlaid (upper) and offset (lower)

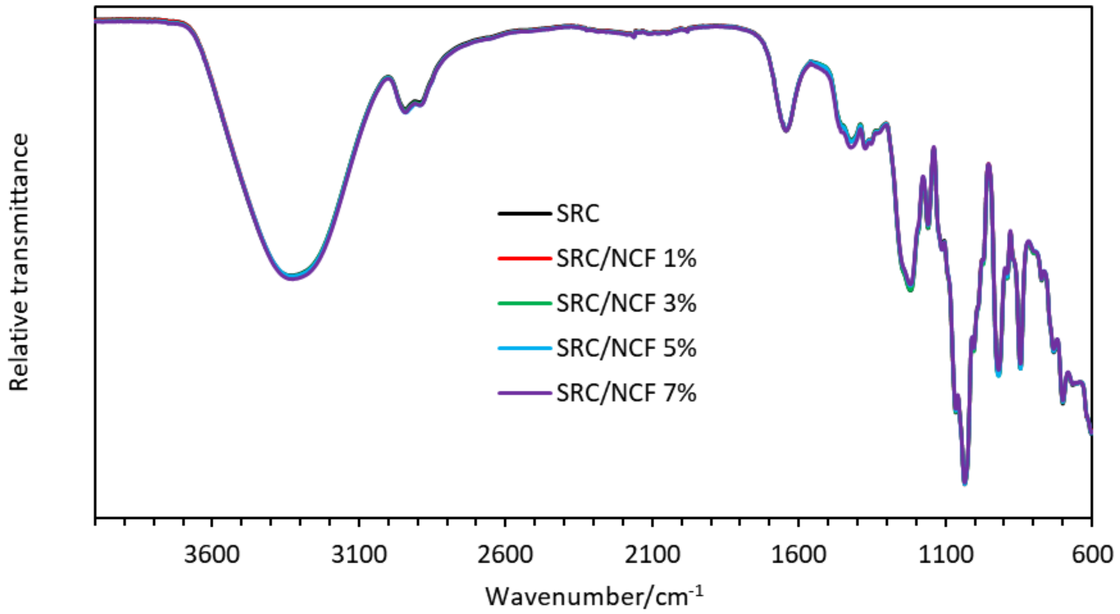

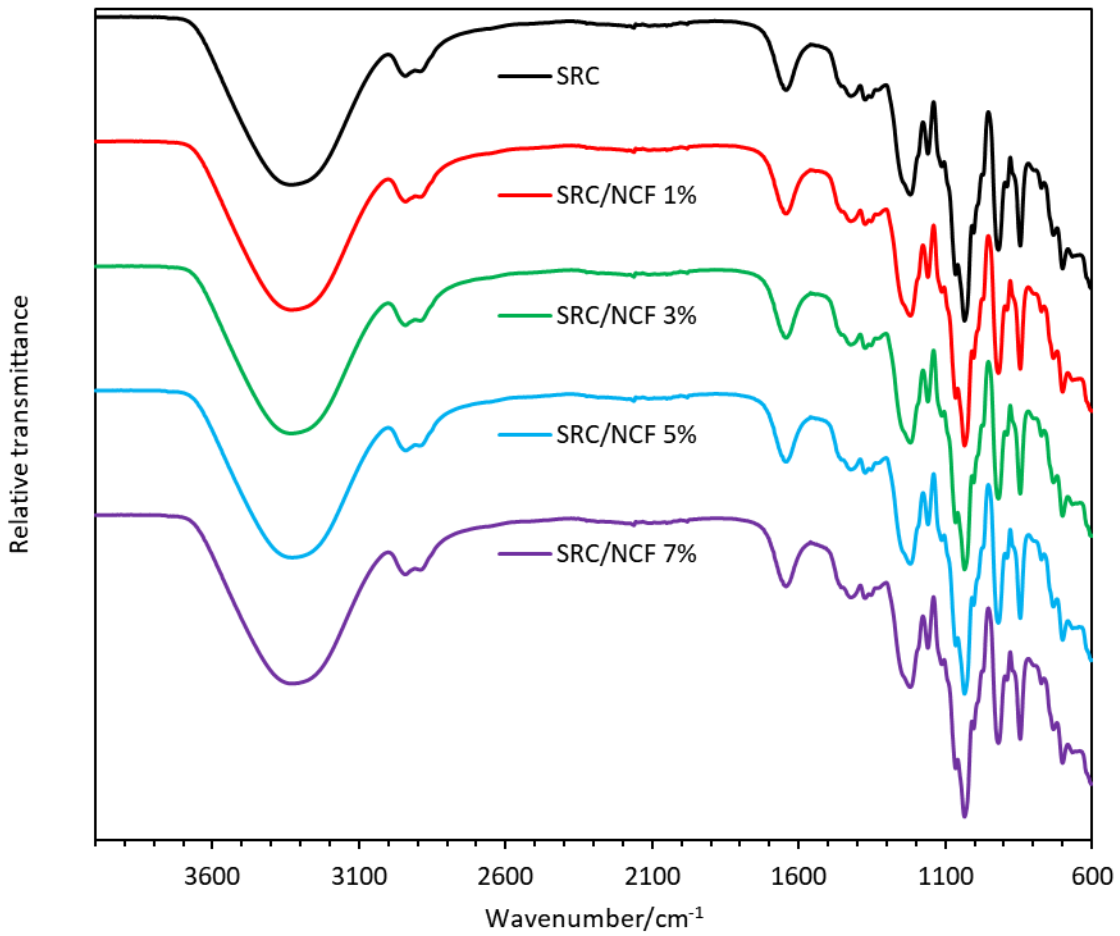

Figure S4. FTIR spectra of the RC/NCF films: overlaid (upper) and offset (lower)

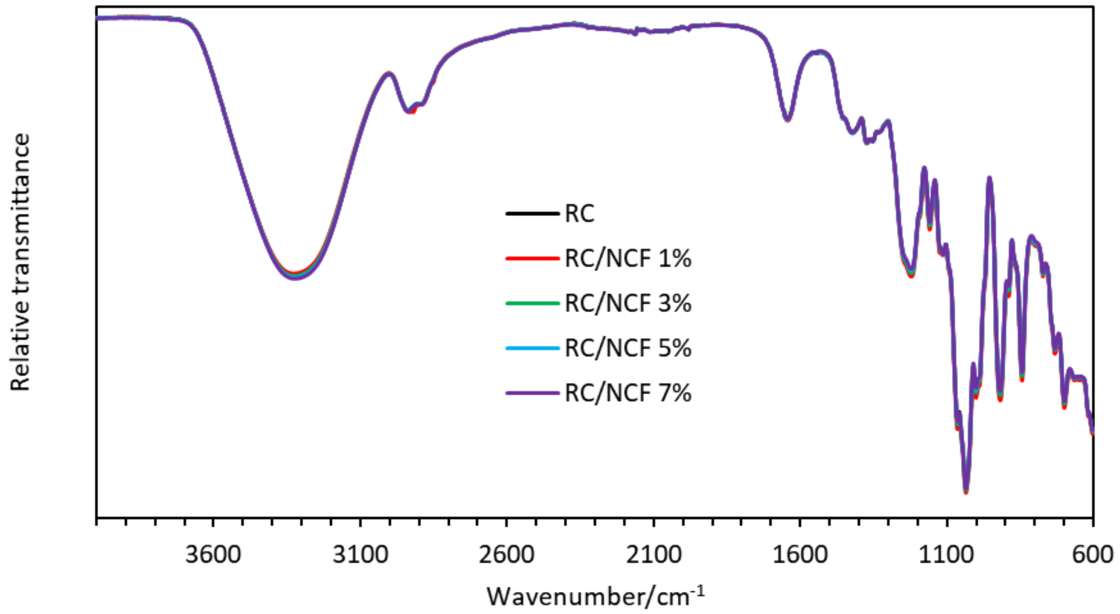

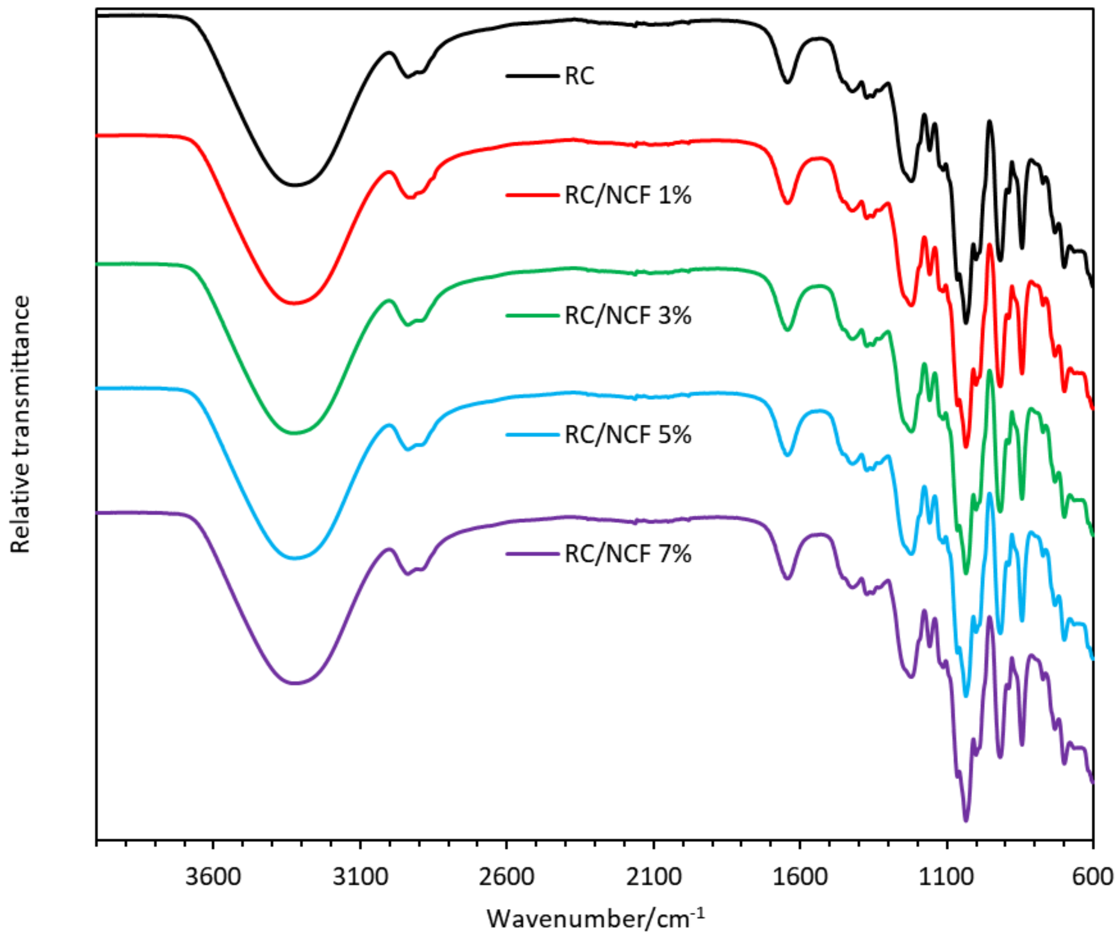

Supplement: Supplementary file 1 [file polymers-12-01145-s001.pdf]
